# Study on the prediction of postoperative metastasis in renal cancer using perirenal fat CT radiomics combined with clinical features

**DOI:** 10.3389/fonc.2026.1766300

**Published:** 2026-02-18

**Authors:** Jing Zhou, Tiantian Zhou, Yuqiong Yang, Cong Zhang, Yichuan Ma, Jiali Xu

**Affiliations:** 1 Department of Radiology, The First Affiliated Hospital of Bengbu Medical University, Bengbu, Anhui, China; 2 School of Graduate, Bengbu Medical University, Bengbu, Anhui, China

**Keywords:** clinical features, computedtomography, perirenal fat, postoperative metastasis, radiomics, renal cancer, TotalSegmentator

## Abstract

**Background:**

This study aims to develop a combined predictive model for predicting postoperative metastasis risk in renal cell carcinoma (RCC) patients based on preoperative arterial-phase Computed tomography(CT) images, integrating clinical data, perirenal fat (PRF), and tumor radiomics features.

**Methods:**

A retrospective analysis was conducted on abdominal CT images and clinical data of patients with pathologically confirmed renal cell carcinoma. Inclusion criteria included preoperative CT scanning, biopsy or surgical confirmation of RCC, and postoperative follow-up to assess metastasis status. Exclusion criteria included patients who had undergone endocrine or anti-tumor treatments. The TotalSegmentator model was used for bilateral PRF segmentation, and radiomics features were extracted. Clinical models, PRF radiomics models, and tumor radiomics models were constructed and integrated into a combined predictive model (Nomogram). The performance of the models was evaluated using receiver operating characteristic (ROC) curves and area under the ROC curve (AUC) values.

**Results:**

A total of 120 patients were included, with 36 (30%) developing postoperative metastasis. The clinical model (AUC = 0.877) identified tumor maximum diameter and neutrophil count as independent predictive factors. The PRF radiomics model (AUC = 0.841) and tumor radiomics model (AUC = 0.848) performed well. The combined model (Nomogram) achieved an AUC of 0.958, significantly outperforming the individual models. All models demonstrated good calibration, and decision curve analysis confirmed their clinical net benefit.

**Conclusion:**

The combined predictive model developed in this study, integrating preoperative clinical data, PRF, and tumor radiomics features, effectively predicts postoperative metastasis risk in RCC patients. This model provides valuable non-invasive information for preoperative metastasis risk assessment and offers reliable guidance for personalized treatment plans, highlighting the critical role of the tumor microenvironment in RCC progression.

## 1 Introduction

Renal cancer is a serious global disease, ranking among the top five in terms of both incidence and mortality. Surgical resection remains the primary curative approach for localized renal cancer, but the postoperative metastasis rate, which can reach 20%–30%, remains a major challenge affecting long-term survival for patients (1). Currently, traditional indicators, such as TNM staging based on postoperative pathology and WHO/ISUP nuclear grading (2), have limited accuracy in predicting individual postoperative metastasis risks and cannot achieve preoperative assessment, thus limiting their practicality in treatment planning. Traditional chemotherapy and radiotherapy are not effective for renal cancer, and there is currently a lack of relatively effective postoperative adjuvant therapies. Literature reports suggest that some “high-risk” patients may benefit from postoperative targeted adjuvant therapy (3), reducing metastasis; however, approximately 50% of patients in this “high-risk” subgroup will not experience metastasis and thus do not require expensive targeted therapy.

In recent years, research on the relationship between perirenal fat (PRF) and renal cancer has increased. PRF has been confirmed to no longer be a simple physical isolation layer but a highly active metabolic and immune organ. According to the American Joint Committee on Cancer staging manual (8th edition), PRF infiltration is an important criterion for pT3a classification, which is closely associated with high metastatic risk (4). Renal cancer cells induce pathological remodeling of perirenal adipocytes by releasing signaling molecules (such as IL-6 and TNF-α), and the latter establishes a proinflammatory, proangiogenic, and proinvasive microenvironment, thus accelerating both local progression and distant metastasis of renal cancer (5, 6). Furthermore, obesity, metabolic disorders, or electrolyte changes may lead to inflammation in the PRF, and this inflammation is associated with increased invasiveness and poor prognosis in renal cancer (7).

Radiomics feature analysis can provide information for detection, risk stratification, and treatment, utilizing images obtained from existing follow-up imaging tools without additional studies (8). Unlike previous studies mainly focusing on tumor radiomics, this research emphasizes the prognostic value of PRF features. By analyzing PRF radiomics, a new perspective on the role of the adipose microenvironment in renal cancer progression is provided. Liu et al. (9) confirmed that magnetic resonance imaging–based prostate periprostatic fat features can predict Gleason score upgrading. In addition, related researchers have found that radiomics features of adipose tissue can effectively predict distant metastasis and prognosis in breast cancer (10). Hu et al. (11) reported that magnetic resonance imaging radiomics features of hepatic cells and peritumoral fat have value in predicting microvascular invasion. These studies consistently indicate that the imaging phenotype of adipose tissue is an important biomarker reflecting tumor invasiveness. Currently, the prediction of postoperative metastasis in renal cell carcinoma primarily relies on the tumour’s own clinical pathological characteristics (such as staging and grading) or intra-tumour radiomics analysis. Research into the imaging features of the tumour microenvironment—perirenal fat (PRF)—has certain limitations. Previous studies have mostly focused on qualitative assessments or quantitative indicators (such as fat thickness) of perirenal fat infiltration, lacking systematic exploration of its deeper imaging phenotypes, such as heterogeneity and textural features (12). No studies have yet comprehensively integrated CT radiomics features of perirenal fat with clinical characteristics to construct multidimensional predictive models. Therefore, this research adopts this approach, aiming to overcome the limitations of traditional predictive frameworks. For the first time, perirenal fat is treated as an independent and significant source of predictive information, revealing its potential association with renal cell carcinoma invasion and metastasis from the perspective of the tumour microenvironment. Innovatively, this study performs multimodal fusion of PRF radiomics features with clinical characteristics, thereby mitigating biases inherent in single-source data while enhancing the model’s biological interpretability and predictive efficacy. The constructed combined predictive model enables non-invasive, convenient preoperative assessment of postoperative metastasis risk, aiding clinicians in formulating personalised follow-up strategies and adjuvant treatment decisions.

Therefore, this study aims to systematically investigate the independent and incremental value of PRF computed tomography (CT) radiomics features in predicting postoperative metastasis in renal cancer. The goal is to develop and validate a combined model integrating clinical features and tumor and PRF radiomics features, with the aim of providing a basis for noninvasive, accurate preoperative risk assessment and ultimately offering individualized postoperative adjuvant treatment strategies for clinicians.

## 2 Materials and methods

### 2.1 Research cohort selection

A retrospective analysis was conducted on abdominal CT images and clinical data of patients with pathologically confirmed renal cell carcinoma. Inclusion criteria: (1) Patients underwent routine abdominal CT scans (both noncontrast and contrast-enhanced) within one month before surgery; (2) renal cancer was confirmed by biopsy or surgical pathology, with regular postoperative follow-up to determine metastasis status; (3) imaging quality met diagnostic requirements; (4) medical records, including imaging and laboratory data, were complete; and (5) age >18 years. Exclusion criteria: (1) Patients who had received endocrine or antitumor therapy before surgery; (2) unclear pathological diagnosis; and (3) incomplete clinical data or loss to follow-up. All pathological diagnoses of renal tumors were made by the Department of Pathology at the First Affiliated Hospital of Bengbu Medical College based on biopsy, partial resection, or radical resection specimens. Patient information was anonymized for this study. Therefore, informed consent was waived for all patients’ guardians, and approval was obtained from the hospital’s Medical Ethics Committee (ethics approval number: No. 153 [2025]), in accordance with the Declaration of Helsinki.

### 2.2 Clinical data

Clinical data and CT parameters included sex, age, side of the affected kidney, maximum tumor diameter (MaxDiameter), albumin-to-globulin ratio, C-reactive protein, lymphocyte count, platelet count, neutrophil count, fibrinogen, neutrophil-to-lymphocyte ratio, platelet-to-lymphocyte ratio, bilateral renal PRF volume, and bilateral renal PRF average density.

### 2.3 Patient follow-up

Follow-up was conducted every 3–4 months in the first year, every 6 months in the second and third years, and annually thereafter. Follow-up included abdominal contrast-enhanced imaging, with the primary endpoint being the occurrence of metastasis or death due to other causes.

### 2.4 CT image acquisition

Imaging data were obtained from a GE 64-slice spiral CT scanner and a GE Revolution 256-slice spiral CT scanner, with scan parameters set to tube voltage 120 kV, tube current 250 mA, and pitch 1.5 mm. Patients were positioned supine, and scanning was performed during breath-hold. The scanning range covered the entire abdomen, with 60–80 ml of iodine contrast agent injected via the antecubital vein at a rate of 3.5 mL/s. Arterial-phase and venous-phase enhanced images were obtained 25–30 seconds and 55–60 seconds after contrast injection, respectively, and then uploaded to the Picture Archiving and Communication System. All studies included three-phase scans: noncontrast, arterial phase, and venous phase.

### 2.5 Image segmentation and radiomics feature extraction

**Figure 1:**
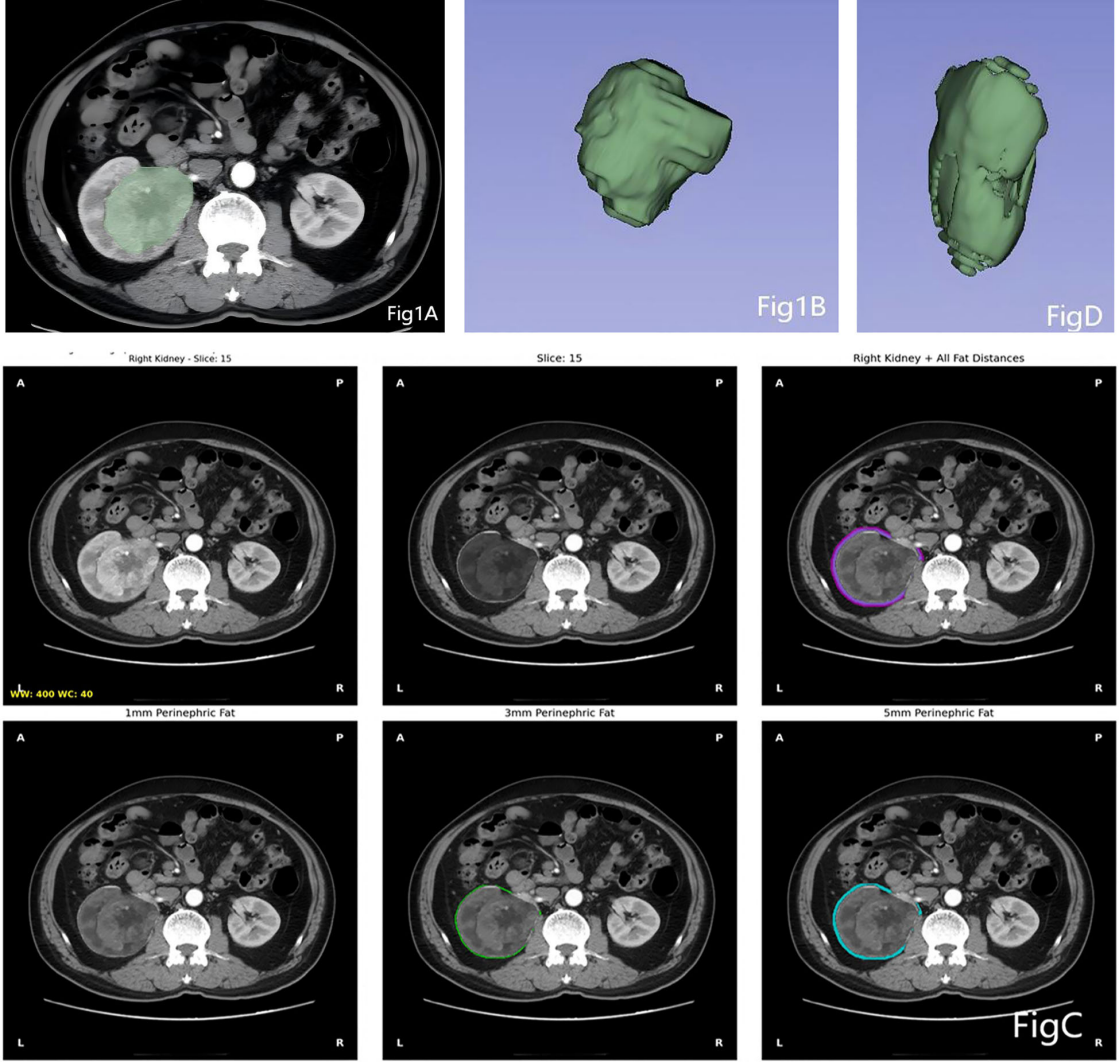
Regions of interest (ROIs) for perirenal fat and tumor on the affected side. **(A)** and **(B)** are ROIs of the tumor; **(C)** represents the perirenal fat segmented within 1mm, 3mm, and 5mm ranges using the TotalSegmentator tool; **(D)** is the ROI of the perirenal fat within the 5mm range.

The images of all patients, after being selected, were exported from the hospital’s Picture Archiving and Communication System in DICOM format, and the arterial-phase axial images of each patient were selected. The arterial phase clearly displays the anatomical features of both the tumor and the PRF tissue. The images were then imported into 3D Slicer (open-source software version 5.4.0, https://www.slicer.org) and converted to NIfTI format. Using the TotalSegmentator model (13) (based on the nnU-Net architecture), bilateral renal segmentation was performed on the original arterial phase CT images (in NIfTI format), resulting in a preliminary binary mask of the kidneys. Subsequently, morphological optimization of the kidney mask was performed to obtain the final kidney label (14). Based on the optimized kidney mask, the Euclidean distance transform was used to calculate the true spatial distance from each voxel to the kidney boundary. Fat tissue was identified based on a standard CT value threshold (15) (–190 to –10 HU). Research (16) indicates that inflammatory infiltration, pro-fibrotic, and pro-angiogenic effects of renal cell carcinoma can extend into adipose tissue surrounding the tumor by several millimeters. A 1mm range may only reflect the most immediate interface effects, whereas a 5mm range is more likely to capture these biologically significant, broader tumor-adipose interaction zones. The radiomics information contained within these zones may be richer and possess greater predictive potential. Fat masks within different distance ranges (1, 3, and 5 mm) from the renal boundary were extracted for both the left and right kidneys, and the PRF region of interest (ROI) within a 5-mm range on the affected side was identified (**Figure 1**). Quantitative metrics, including fat volume, visual preview images, and summary data tables, were automatically generated.

For the tumor Region of Iinterst (ROI), the original CT images were imported into 3D Slicer (open-source software version 5.4.0, https://www.slicer.org) for renal tumor segmentation. A semi-automatic tool was used to manually segment tumor examples (**Figure 1**), and ROIs were outlined semi-automatically along the tumor boundary in the arterial-phase axial images. Traditional imaging features were measured while avoiding areas of peritumoral edema, necrosis, cystic components, and calcification. The tumor’s contour in the CT axial images was reviewed and corrected by a radiologist with 10 years of clinical experience.

Radiomics features of both the tumor and affected PRF were extracted using Pyradiomics, including shape-based features, first-order statistics, gray-level co-occurrence matrix, gray-level dependence matrix, gray-level run-length matrix, gray-level size zone matrix, neighboring gray-level difference matrix, wavelet transform, and Laplacian transform. A total of 1680 radiomics features were extracted from each ROI.

### 2.6 Model construction

#### 2.6.1 Clinical feature model

The clinical model was divided into training and testing sets at a 7:3 ratio. Binary multivariate logistic regression, initially using univariate analysis followed by multivariate analysis, was performed to identify independent risk factors using the Akaike Information Criterion (AIC) and basic event rule statistical methods. Tenfold cross-validation was performed in the training cohort to determine the optimal hyperparameters, and internal validation was conducted using the independent test set. The odds ratio (OR), 95% confidence interval (CI), and P-value were calculated.

#### 2.6.2 Radiomics model

In this study, the data were divided into training and testing sets at a 7:3 ratio. Sex and age from the clinical data were used as references to ensure that there were no significant differences in clinical characteristics between the two groups after the division. To address the imbalance in the training dataset, the Synthetic Minority Over-sampling Technique was applied for balancing. The following method is employed for processing features in both the training and test sets. First, Z-score normalization was performed on the feature matrix, followed by dimensionality reduction using Pearson’s similarity. The PRF model applied the recursive feature elimination algorithm to select features, which selects subsets of data and recursively identifies relevant features based on the labels. A support vector machine classifier was used. For the tumor model, the Analysis of Variance algorithm was used for feature selection, and logistic regression was employed as the classifier. Both models underwent tenfold cross-validation in the training cohort to determine the optimal hyperparameters, followed by internal validation using the independent test set.

Based on the clinical feature model (Model 1), affected-side PRF radiomics model (Model 2), and tumor radiomics model (Model 3), multivariable binary logistic regression analysis was conducted to build a combined diagnostic model and nomogram (Model 4).

### 2.7 Statistical analysis

Categorical variables were analyzed using the chi-square test. Continuous variables with a normal distribution were compared using the independent t-test and expressed as mean ± standard deviation (SD). Continuous variables with a non-normal distribution were analyzed using the Mann–Whitney U test and expressed as P50 (P25, P75). Multiple metrics were used to evaluate the model performance, including the receiver operating characteristic (ROC) curve and the calculation of the area under the curve (AUC) to quantify diagnostic effectiveness. The accuracy (ACC), standard error (SE), sensitivity (Sen), specificity (Spec), positive predictive value, negative predictive value, and Youden Index were also calculated. The OR for each variable in the clinical model was calculated to quantify its impact on the outcome. The DeLong test was used to compare the AUC of any two models, and the Hosmer–Lemeshow test was conducted for the nomogram. Calibration curves for all four models were drawn to visually represent the prediction performance. Decision curves were used to evaluate the net benefit of each model. All statistical analyses were performed using R software (version 4.3.3), with a two-sided P-value of <0.05 considered statistically significant.

## 3 Results

### 3.1 Patient characteristics

A total of 120 patients were included in the study. Based on postoperative follow-up results, they were divided into a metastasis group (36 cases (30%)) and a nonmetastasis group (84 cases (70%)). Based on age and gender data from clinical records, subjects were randomly allocated in a 7:3 ratio to form a training set comprising 84 cases and a test set comprising 36 cases. All patients underwent either partial or radical nephrectomy. Of the patients, 68 were male (56.7%) and 52 were female (43.3%). The majority of patients were aged between 50 and 70 years, with 53.3% (64/120) of tumors located in the left kidney. A comparison of baseline data between the two groups is shown in **Table 1**. No significant differences were observed in the clinical data or CT parameters of healthy-side PRF between the training and test sets (P > 0.05 for all).

**Table 1:** Comparison of baseline data betweentraining set and test set.

Characteristic	Test set^a^ n = 36	Training set^a^ n = 84	P-value^b^
Sex (%)			0.968
female	15 (41.7%)	37 (44.0%)	
male	21 (58.3%)	47 (56.0%)	
Age (median [IQR])	58 (52,67)	59 (53,68)	0.636
Affectedside (%)			0.281
left	16 (44.4%)	48 (57.1%)	
right	20 (55.6%)	36 (42.9%)	
MaxDiameter (median [IQR])	6.60 (5.73,7.64)	6.54 (5.96,7.52)	0.966
AG (median [IQR])	1.70 (1.40,1.90)	1.60 (1.40,2.00)	0.548
CRP (median [IQR])	3.63 (3.21,5.00)	3.81 (3.11,5.39)	0.6
Lcell (mean (SD))	2.01 ± 0.64	1.84 ± 0.69	0.2
Plt (mean (SD))	249 ± 70	229 ± 61	0.2
Ncell (mean (SD))	4.32 ± 2.52	3.97 ± 1.39	0.9
FIB (median [IQR])	3.57 (2.72,4.05)	2.88 (2.61,3.38)	0.27
NLR (mean (SD))	2.92 ± 0.91	2.87 ± 1.07	0.806
PLR (mean (SD))	160 ± 57	159.60 ± 59.79	0.941
Fat_Volume (cm3)(median [IQR])	63 (52,73)	69.18 (55.40,78.73)	0.251
Fat_Density (HU)(median [IQR])	-72 (-78,-69)	-75.07 (-81.07,-65.50)	0.584

a, Mean ± SD, Median (Q1, Q3); b, Wilcoxon rank sum test.

### 3.2 Clinical feature model (Model 1)

**Figure 2:**
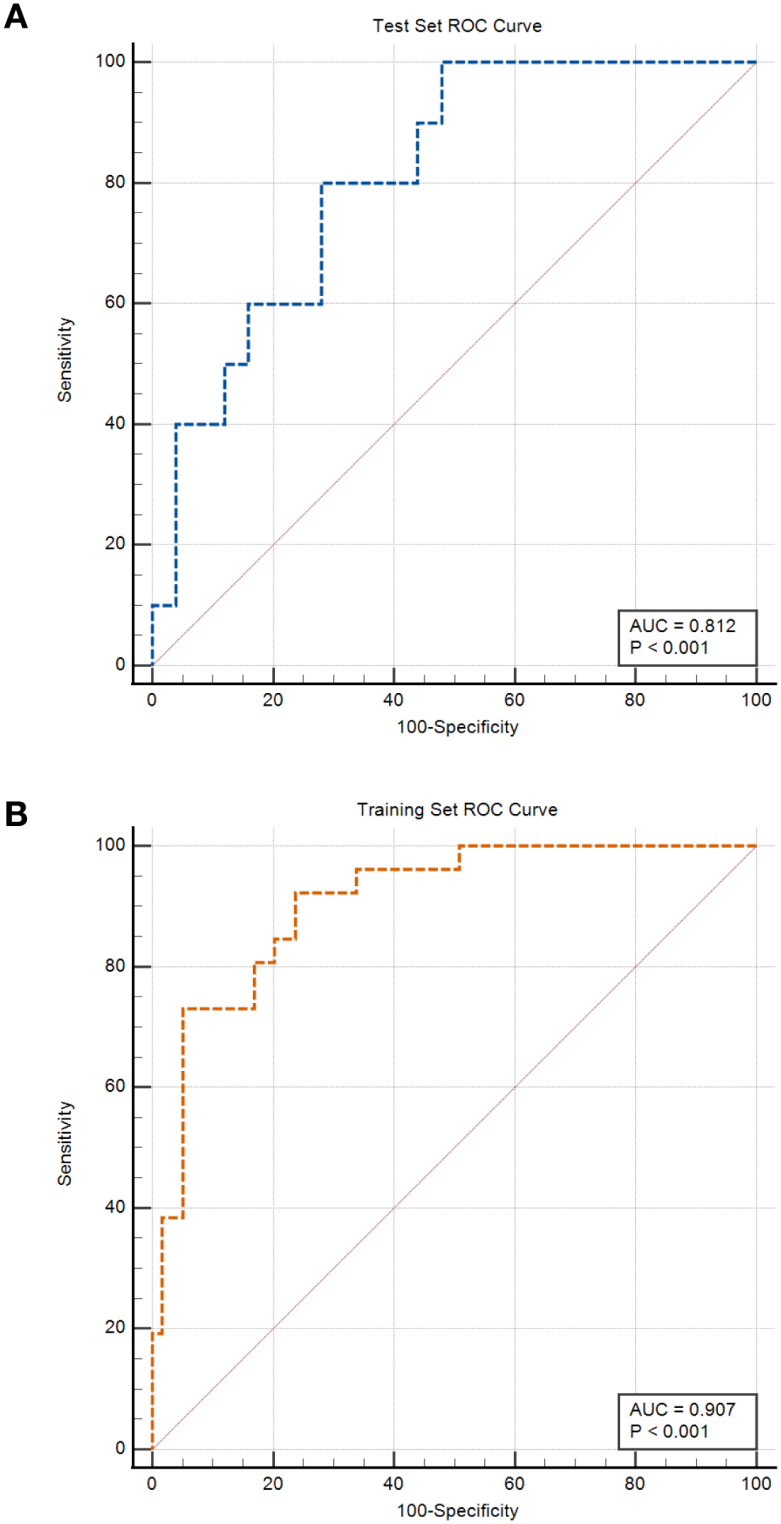
Clinical feature model training and test set ROC curves. **(A)** represents the ROC curve for the training set; **(B)** represents the ROC curve for the test set.

Postoperative metastasis status was used as the dependent variable, with clinical data and CT parameters of bilateral PRF (15 indicators in total) as independent variables. A binary multivariate logistic regression analysis was performed to establish the regression model, identifying independent factors, OR, and 95% CIs for distinguishing postoperative metastasis in patients with renal cancer (**Table 2**). After multivariate analysis, MaxDiameter and Ncell were identified as independent risk factors for predicting metastasis (OR = 2.44 and 2.40, respectively). ROC analysis determined that the optimal cutoff value for maximum tumor diameter was 6.99 cm, and for neutrophil count, it was 4.3×10^9^/L. The clinical model achieved an AUC of 0.907 (95% CI: 0.825–0.960, Sen = 0.923, Spec = 0.762) in the training set ROC curve (**Figure 2**). In the test set, the model’s ROC curve achieved an AUC of 0.812 (95% CI: 0.644–0.924, Sen = 0.892, Spec = 0.787). Finally, for the entire dataset (n = 120), the AUC reached 0.877 (SE = 0.032, 95% CI: 0.805–0.930) (**Table 3**).

**Table 2:** Univariate and multivariate analysis of clinical characteristics and CT parameters of perirenal fat on the contralateral side in patients from the training and test sets.

Parameter	Univariate analysis			Multivariate analysis		
	OR	95%CI	P	OR	95%CI	P
Sex	0.87	0.34-2.22	0.77			
Age	1.06	1.00-1.11	0.38			
Affectedside	1.02	0.40-2.56	0.97			
MaxDiameter*	2.16	1.45-3.21	<0.01	2.44	1.60-4.17	0.0002
AG*	0.06	0.01-3.21	<0.01			
CRP*	1.78	1.27-2.5	<0.01			
Lcell	0.40	0.19-0.85	0.02			
Ncell*	2.02	1.37-2.97	<0.01	2.40	1.54-4.12	0.0004
Plt	1.01	1.00-1.02	0.12			
FIB*	3.14	1.60-6.16	<0.01			
NLR*	2.80	1.55-5.05	<0.01			
PLR	1.01	1.00-1.02	0.07			
Fat_Volume(cm^3^)	0.98	0.95-1	0.04			
Fat_Density(HU)	1.02	0.99-1.05	0.24			

AG, Albumin-to-Globulin Ratio; CRP, C-reactive protein; Lcell, Lymphocyte count; Ncell, Neutrophil count; Plt, Platelet count; FIB, Fibrinogen; NLR, Neutrophil-to-Lymphocyte Ratio; PLR, Platelet-to-Lymphocyte Ratio.

**Table 3:** Diagnostic effectiveness of four prediction models.

Variable	AUC	SE*	95% CI #
tumor_radiomics	0.849	0.0399	0.772 to 0.907
Clinical_indicators	0.877	0.0316	0.805 to 0.930
fat_radiomics	0.837	0.0425	0.759 to 0.898
union_model	0.958	0.0158	0.905 to 0.986

*De-Long et al.1988; #Binomial exact; SE, Standard Error; CI, Confidence Interval.

### 3.3 Affected-side PRF radiomics model (Model 2)

**Figure 3:**
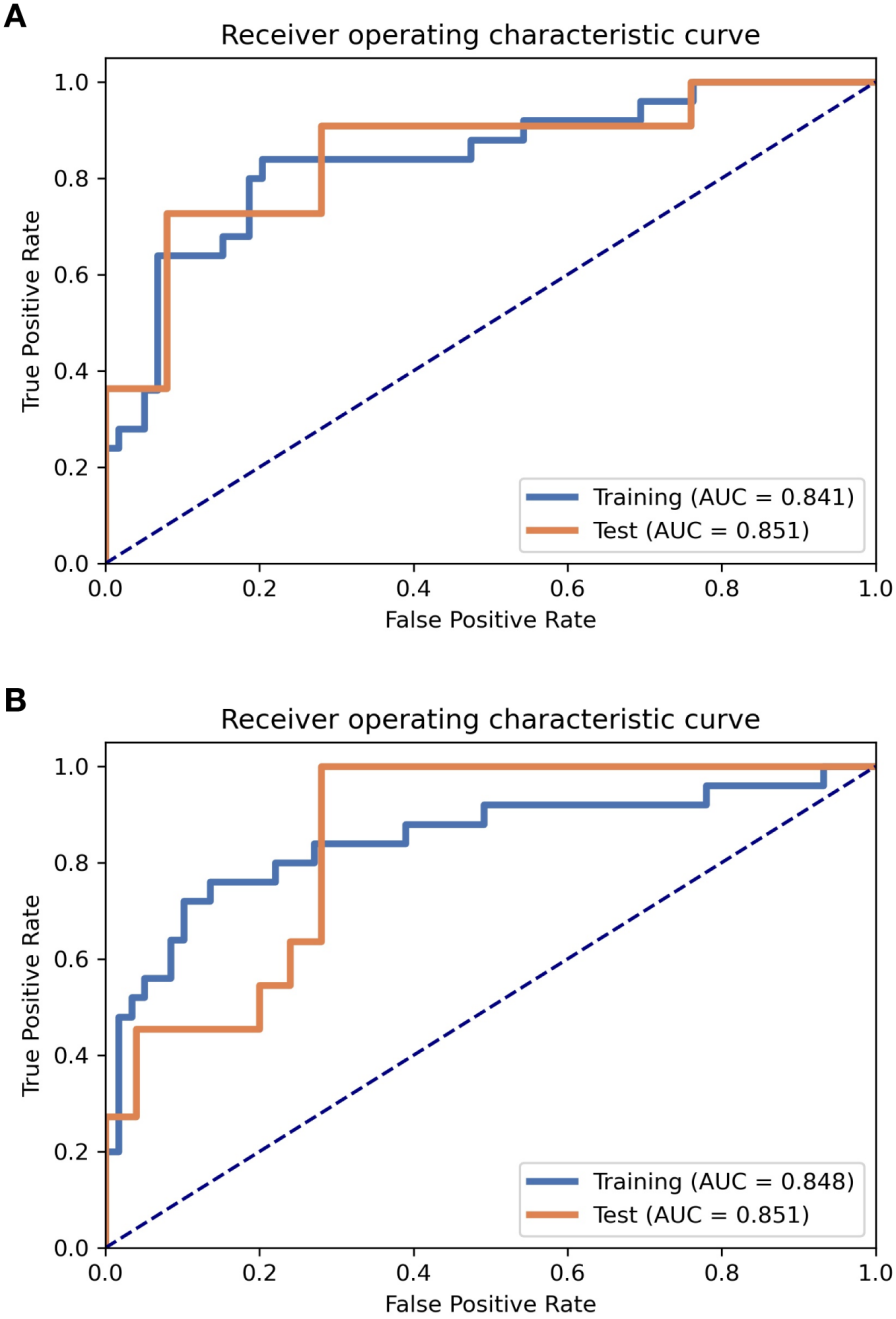
ROC curves for PRF and tumor radiomics models on the training and test sets. **(A)** PRF radiomics model; **(B)** tumor radiomics model.

A model was constructed based on the five most valuable features. The model performed excellently in the training set, achieving an AUC of 0.841 and an ACC of 0.810. In the test set, the model’s generalization ability was validated, with an AUC of 0.851 and an ACC of 0.861 (**Table 4**). For the entire dataset (n = 120), the model achieved an AUC of 0.837 (SE = 0.043, 95% CI: 0.759–0.898) (**Table 3**). The prediction model’s Sen, Spec, positive predictive value, negative predictive value, Youden Index, and SD were calculated for both the training and test sets, with detailed results shown in **Table 4**. ROC curve results for the PRF model in the training and test sets are shown in **Figure 3**.

**Table 4:** Diagnostic effectiveness of fat and tomor raidomics prediction models in train and test set.

Indicators	Value* (fat_radiomics)		Value* (tumor_radiomics)	
	Training set	Test set	Training set	Test set
Accuracy	0.8095	0.8611	0.8333	0.8056
AUC	0.8407	0.8509	0.8481	0.8509
AUC 95% CI	[0.7427-0.9386]	[0.7004-1.0000]	[0.7440-0.9523]	[0.7262-0.9656]
NPV	0.9216	0.8846	0.8947	0.9091
PPV	0.6364	0.8000	0.7037	0.6111
Sen	0.8400	0.7273	0.7600	0.8297
Spec	0.7966	0.9200	0.8644	0.7200
Youden Index	0.6366	0.6473	0.6244	0.7200
Std	0.05	0.0768	0.0531	0.0636

AUC, Area Under Curve; CI, Confidence Interval; NPV, Negative Predictive Value; PPV, Positive Predictive Value; Sen, Sensitivity; Spec, Specificity; Std, Standard; *FeAture Explorer(FAE).

### 3.4 Tumor radiomics model (Model 3)

The model based on 13 of the most valuable features achieved the highest AUC on the validation dataset. The AUC and ACC were 0.848 and 0.833, respectively. In the test dataset, the model achieved an AUC of 0.851 and an ACC of 0.806 (**Table 4**). For the entire dataset (n = 120), the model achieved an AUC of 0.840 (SE = 0.040, 95% CI: 0.772–0.907) (**Table 3**). The prediction model’s Sen, Spec, positive predictive value, negative predictive value, Youden Index, and SD were also calculated for both the test and training sets, with results shown in **Table 4**. ROC curve results for the tumor model in the training and test sets are shown in **Figure 3**.

### 3.5 Diagnostic performance of the combined model and comparison between models

**Figure 4:**
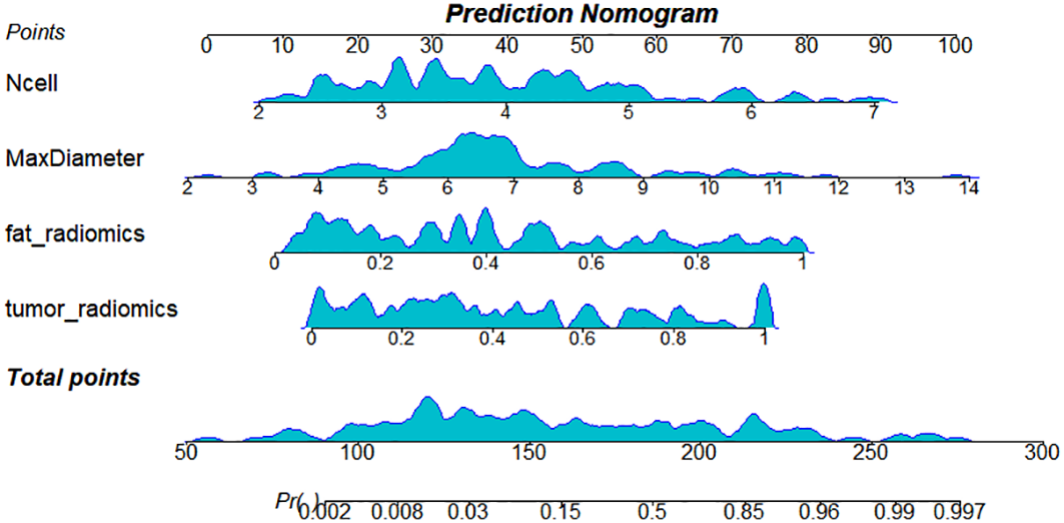
Normangram of the combined model constructed from three models.

**Figure 5:**
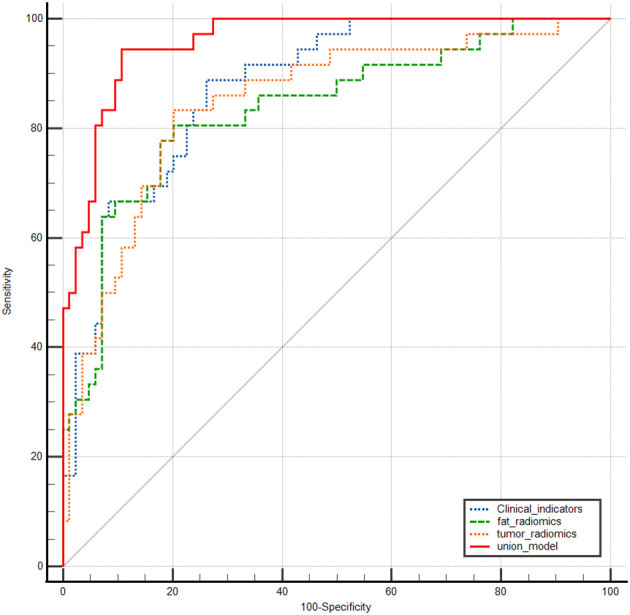
ROC Curves for four predictive models used to distinguish metastatic from Non-metastatic cases.

**Figure 6:**
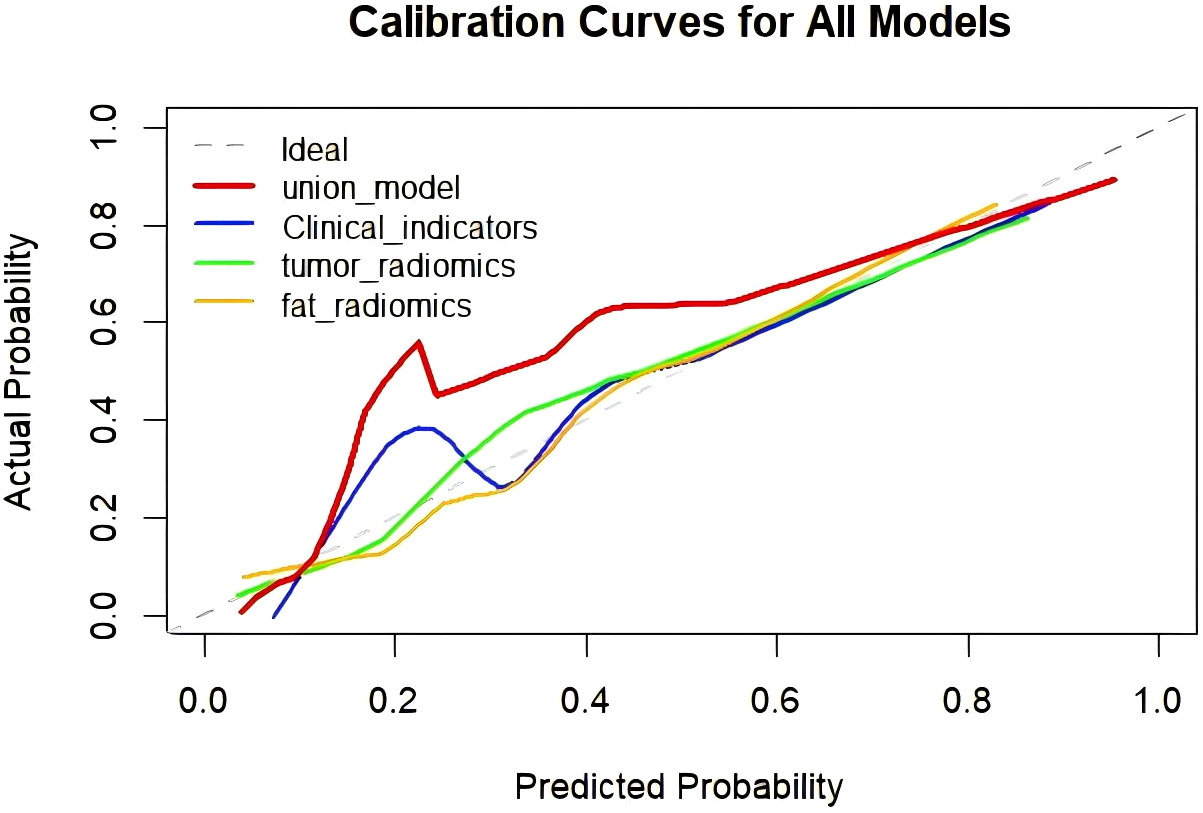
Calibration curves for four predictive models used to distinguish metastatic from Non-metastatic cases.

**Figure 7:**
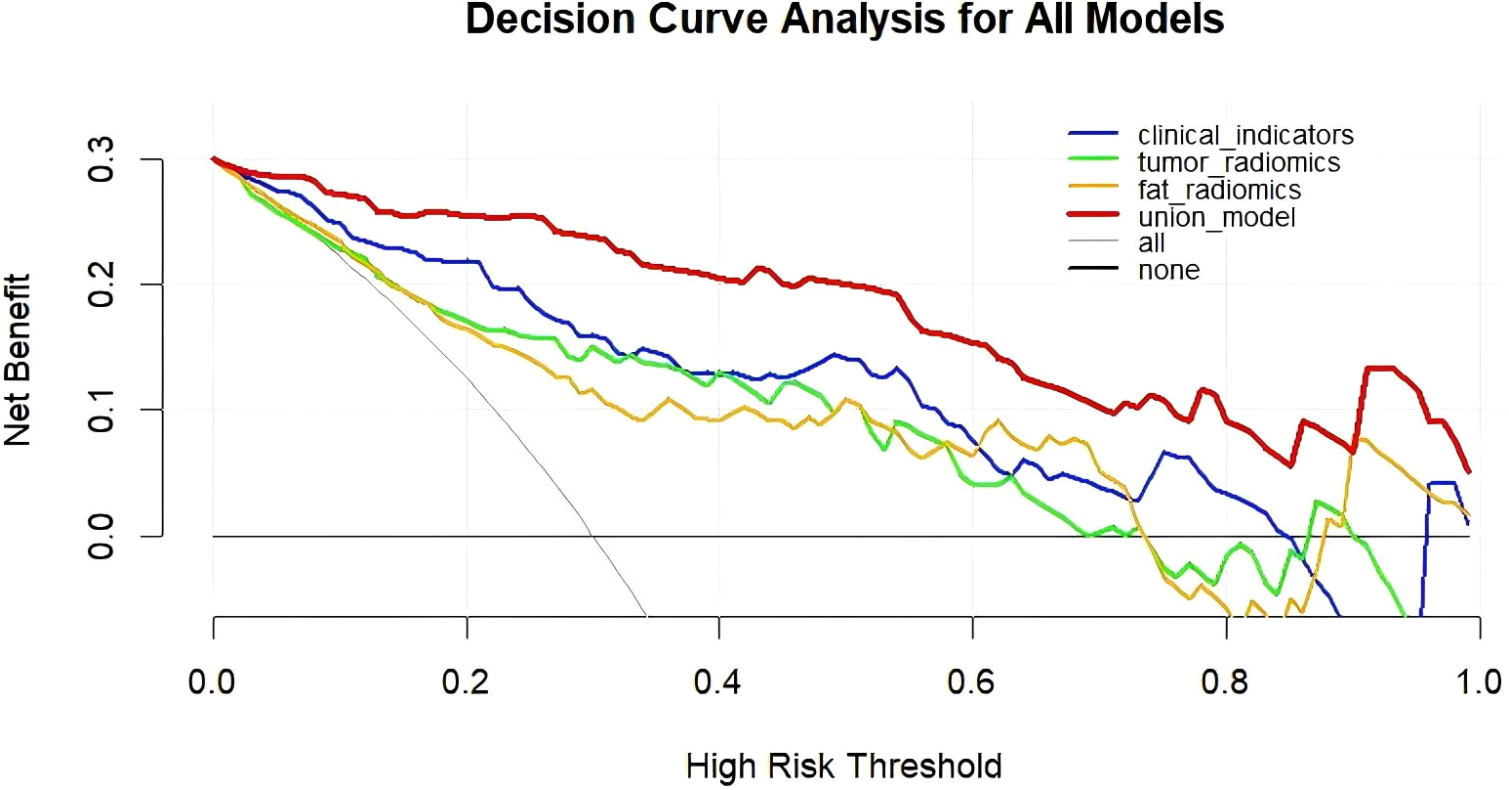
Four rredictive models distinguishing metastatic and Non- metastatic DCA Curves.

Integrate clinical characteristics, PRF, and tumor radiomics features to obtain the predicted probability values from each individual model, thereby constructing a combined diagnostic model—the Nomogram. (**Figure 4**). Based on the entire dataset (n = 120), the combined model achieved an AUC of 0.958 (SE = 0.0158, 95% CI: 0.905–0.986) (**Table 3**). The individual models 1, 2, and 3 had AUC values of 0.877, 0.837, and 0.849, respectively. A comparison of the ROC curves for the four prediction models is shown in **Figure 5**. The combined model (Model 4) demonstrated the best diagnostic performance. The Hosmer–Lemeshow test and calibration curves (**Figure 6**) showed good consistency between the predicted and observed values for all four models. The DeLong test results indicated no significant statistical differences in AUC values between Model 1 and Model 2, Model 1 and Model 3, or Model 2 and Model 3 (P > 0.05). However, the AUC of the combined model (Model 4) was significantly higher than that of Models 1, 2, and 3 (P < 0.05 for all) (**Table 5**). Decision curve analysis (DCA) further demonstrated that all four models provided clinical net benefits (**Figure 7**).

**Table 5:** Cross-validation of diagnostic performance among four predictive models.

Model categories	Difference between areas	SE*	95% CI	z statistic	Significance level P value
Clinical_indicators~ fat_radiomics	0.0400	0.0569	-0.0715 to 0.152	0.703	0.4820
Clinical_indicators~ tumor_radiomics	0.0288	0.0517	-0.0725 to 0.130	0.557	0.5776
Clinical_indicators~ union_model	0.0810	0.0282	0.0258 to 0.136	2.873	0.0041
fat_radiomics~ tumor_radiomics	0.0112	0.0521	-0.0909 to 0.113	0.216	0.8292
fat_radiomics~ union_model	0.121	0.0394	0.0437 to 0.198	3.069	0.0021
tumor_radiomics~ union_model	0.110	0.0349	0.0415 to 0.178	3.149	0.0016

*DeLong et al., 1988; SE, Standard Error; CI, Confidence Interval.

AUC, area under the curve; PRF, perirenal fat; CT, computed tomography; DCA, decision curve analysis; SE: Standard Error; CI: Confidence Interval; ROC, receiver-operating characteristic; ROI, region of interest.

## 4 Discussion

This study aimed to systematically assess the value of clinical features, affected-side PRF radiomics features, tumor radiomics features, and the combined use of these factors in predicting postoperative metastasis risk in patients with renal cancer. We sequentially constructed the clinical model (Model 1), affected-side PRF radiomics model (Model 2), tumor radiomics model (Model 3), and a combined model (Model 4) integrating the first three. The results showed that each of the three individual models demonstrated some predictive ability (AUCs of 0.877, 0.837, and 0.840 for Models 1, 2, and 3, respectively). However, the combined model (Model 4), which integrated multidimensional information, achieved the optimal discriminatory performance in both the training and validation sets, with an AUC as high as 0.958. This performance was significantly superior to that of any single model. These findings suggest that integrating macroscopic clinical indicators, the tumor’s microenvironment, and its inherent heterogeneity allows for more precise preoperative, noninvasive prediction of postoperative metastasis risk in renal cancer.

In advanced-stage renal cancer, the recurrence and metastasis rates are relatively high, with a recurrence rate of 20%–30% after surgery. If combined with PRF infiltration, the postoperative recurrence rate increases to 40% (17). An increasing body of research suggests that in renal cell carcinoma, the thickness of PRF is positively correlated with tumor stage and negatively correlated with prognosis. Additionally, PRF can promote an inflammatory microenvironment, thereby exacerbating chronic kidney disease and metabolic abnormalities. These findings highlight the clinical significance of PRF as a diagnostic and prognostic biomarker, as well as a potential therapeutic target (18). Studies have shown that renal cancer cells can induce the browning of adjacent PRF tissue, which secretes lactate to promote tumor growth, invasion, and metastasis (19). Du et al. (20) indicated that the percentage of PRF is an independent predictor of postoperative mortality risk in renal cancer. Researchers have suggested that for patients at high risk of postoperative recurrence and metastasis (e.g., those with peritumoral fat infiltration), the timely use of targeted and immune adjuvant therapy can significantly reduce the recurrence and metastasis rate. This provides a theoretical basis for the present study. For the clinical model (Model 1), we conducted an extensive evaluation of various clinical variables to predict postoperative metastasis. Multivariable logistic regression analysis was used to identify independent clinical risk factors. The AIC was applied to balance model fit and complexity. The formula for AIC is AIC = 2k − 2ln(L), where k is the number of variables in the model and L is the likelihood function value. The lower the AIC value, the better the model, allowing for the selection of a variable combination that balances fit and simplicity. Additionally, due to the relatively small sample size in this study (n = 120), logistic regression analysis incorporated the basic event number rule to limit the number of variables, ensuring that each variable had sufficient sample support and improving the model’s external validity. The clinical model developed in this study identified tumor maximum diameter and peripheral neutrophil count as independent preoperative predictors. The model achieved an AUC of 0.877 (SE = 0.032, 95% CI: 0.805–0.930) based on the entire dataset (n = 120). This result is consistent with previous studies. For instance, Li et al. (21) confirmed that tumor size is an independent predictor of specific survival in renal cancer, with each additional centimeter of tumor diameter increasing the risk of death. Furthermore, systemic inflammation has been recognized as the “seventh hallmark” of cancer. Templeton et al. (22) clearly pointed out that high neutrophil counts are closely associated with poor overall and cancer-specific survival in various solid tumors, including renal cancer. The underlying mechanism may involve neutrophils promoting angiogenesis and suppressing adaptive immune responses. The results of this study further reinforce the significant value of these indicators in the prognostic evaluation of renal cancer.

CT has become an indispensable imaging modality in the diagnosis and treatment of renal cancer and other tumours. In addition to assessing the kidneys and renal tumors, CT provides rich data on body composition (23). Researchers indicate that unenhanced CT textural analysis can differentiate between pancreatic adenocarcinoma and pancreatic ductal adenocarcinoma, suggesting that CT examinations hold significant importance in tumour diagnosis and differential diagnosis (24). Studies have shown that arterial phase imaging provides optimal contrast for delineating tumour margins, exhibits highest sensitivity in reflecting the microvascular environment of perirenal fat, and demonstrates high concordance with routine clinical examinations (25). Therefore, this study selected axial images from the arterial phase as the raw images for ROI segmentation. For the PRF ROI, the study utilized the TotalSegmentator model (13) (based on the nnU-Net architecture) to perform bilateral kidney segmentation on the raw CT images (in NIfTI format). This model demonstrated high segmentation accuracy in validation (Dice coefficient >0.90). Subsequently, convex hull filling, iterative dilation, and hole filling were applied. Euclidean distance transform was used to calculate the real spatial distance of each voxel to the kidney boundary, using the formula(: (d(p) = \min \sqrt{((px − kx)^2 \cdot sx^2 + (py − ky)^2 \cdot sy^2 + (pz − kz)^2 \cdot sz^2)}), where ((sx, sy, sz)) are the voxel spacings, considering anisotropy. Fat tissue was identified based on standard CT value thresholds (15) (−190 to −10 HU). PRF masks within distance ranges (5 mm) from the left and right kidneys were extracted according to a preset distance interval (16). Finally, bilateral 5-mm PRF ROIs were obtained, along with the PRF volume and average density. Both the bilateral PRF volume and average density were incorporated as CT parameters in the multivariable logistic regression analysis.

Currently, CT-based radiomics predictive models have been extensively studied in various aspects of renal cancer, including differential diagnosis (26), pathological subtyping and grading (27), microvascular invasion (28), and postoperative survival (29). Khene et al. (30) used a combination of radiomics features and a clinical model to predict recurrence after surgical resection of high-risk localized renal cell carcinoma. The model achieved an AUC of 0.81 (95% CI: 0.76–0.85) in the training set and an iAUC of 0.78 (95% CI: 0.69–0.88) in the test set, demonstrating good predictive performance. Relevant studies indicate that CT radiomics can effectively predict lymph node metastasis in pancreatic cancer, thereby providing the theoretical basis for this research (31). Li et al. (32) used TransUNet for automatic segmentation of the kidney and visceral fat tissue and combined PRF and tumor radiomics features with machine learning, achieving accurate predictions for pathological grading of clear-cell renal cell carcinoma. Building on these findings, this study extracted radiomics features from both the renal tumor and PRF. It developed models for the affected-side PRF radiomics (Model 2), tumor radiomics (Model 3), and a combined model (Model 4). In this study, the PRF radiomics model showed excellent performance in the training set, with an AUC of 0.841 (ACC = 0.810, 95% CI = 0.7427–0.9386; Sen = 0.840; Spec = 0.797; Youden Index = 0.637; SD = 0.05). In the test set, the model’s generalizability was validated, with an AUC of 0.851 (ACC = 0.861; 95% CI = 0.7004–1.0000; Sen = 0.727; Spec = 0.920; Youden Index = 0.647; SD = 0.076). Furthermore, the tumor radiomics model achieved an AUC of 0.848 in the training set (ACC = 0.833; 95% CI = 0.7440–0.9523; Sen = 0.7600; Spec = 0.8644; Youden Index = 0.624; SD = 0.0531) and an AUC of 0.851 in the test set (ACC = 0.806; 95% CI = 0.7262–0.9656; Sen = 0.8297; Spec = 0.7200; Youden Index = 0.7200; SD = 0.0636). Both the PRF and tumor models demonstrated an accuracy greater than 0.8, indicating that the predictive models had excellent diagnostic performance. Finally, this study integrated clinical features, PRF, and tumor radiomics features to construct a nomogram model (Model 4). The combined model achieved an AUC of 0.958 (SE = 0.0158, 95% CI: 0.905–0.986) based on the entire dataset (n = 120). The DeLong test indicated that the nomogram model outperformed the PRF, tumor, and clinical models (P < 0.05 for all). The calibration curve showed that the predicted values from all four models were in good agreement with the observed values, and the DCA curve confirmed that all models provided clinical net benefits, with the nomogram model demonstrating the best performance. Researchers employed a CT-based radiomics nomogram to preoperatively distinguish serous cystadenomas from mucin-secreting pancreatic cystadenomas, demonstrating the nomogram’s favourable clinical utility. This finding is consistent with the results of the present study (33). Similarly, Liu et al. (9) combined periprostatic fat, prostate cancer, and clinical data in a nomogram to predict the upgrading of the Gleason score from biopsy to radical prostatectomy, showing good performance. This approach provided a reliable noninvasive tool for Gleason score upgrading risk stratification, which could reduce overtreatment and undertreatment in patients with prostate cancer, aligning with the direction of this study focused on urological diseases (renal cancer) prognosis. The combined model and nomogram developed in this study also demonstrated excellent predictive performance for postoperative metastasis risk in renal cancer (AUC = 0.958).

Numerous studies have shown (4, 32, 34, 35) that in renal cancer, CT or magnetic resonance imaging can be used to observe morphological changes such as blurring or infiltration of the PRF to assist in staging diagnoses of T3a renal cancer. Other studies have focused on quantifying PRF parameters (such as CT values and area) to differentiate between benign and malignant lesions and assess tumor malignancy. Radiomics features of PRF have been used to predict PRF invasion and assist in tumor pathological grading. Beşler et al. (36) found that high PRF density was associated with progression-free survival and confirmed its predictive value for tumor recurrence through ROC analysis. However, this study only used PRF CT values as the core predictive indicator and did not incorporate traditional prognostic factors such as tumor size or clinical data, which limited its ability to fully reflect the complex mechanisms of tumor progression. Therefore, in this study, by analyzing PRF radiomics features and combining them with clinical data and tumor radiomics features, we constructed both single- and combined prediction models (nomogram model) that improve the accuracy of noninvasive preoperative prediction of postoperative metastasis risk in patients with renal cancer. The joint model achieves complementary information, noise cancellation, and a more complete simulation of transferred biological processes through multidimensional feature fusion. The combined model showed the best diagnostic performance. This approach provides clinicians with a tool for accurately identifying patients at high risk of postoperative metastasis and for making individualized decisions regarding adjuvant treatment (e.g., targeted therapy, immunotherapy).

The strength of this study lies in the integration of PRF and renal tumor radiomics features, along with key clinical variables, which helps provide a more comprehensive and accurate prediction of postoperative metastasis. The nomogram developed in this study offers a user-friendly, quantifiable, and personalized predictive tool, which can assist clinicians in making informed decisions about patient management, potentially reducing overtreatment and undertreatment.

## 5 Conclusion

This study demonstrates that preoperative arterial-phase CT imaging-based clinical, PRF radiomics, and tumor radiomics models effectively predict postoperative metastasis risk in patients with renal cancer. Moreover, PRF radiomics features offer incremental predictive value independent of tumor characteristics and clinical parameters. Therefore, the combined prediction model, integrating these multidimensional data, exhibits superior predictive performance compared to any individual model. This model highlights the significant role of the tumor microenvironment in renal cancer progression and provides a more reliable basis for clinical individualized risk stratification and treatment decisions, helping to avoid undertreatment or overtreatment.

## Data Availability

The original contributions presented in the study are included in the article/supplementary material. Further inquiries can be directed to the corresponding author.
